# Dysregulated adaptive immune response contributes to severe COVID-19

**DOI:** 10.1038/s41422-020-0391-9

**Published:** 2020-08-05

**Authors:** Kuai Yu, Jingjing He, Yongjian Wu, Baosong Xie, Xuefei Liu, Bo Wei, Haibo Zhou, Bingliang Lin, Zhixiang Zuo, Wen Wen, Wenxiong Xu, Bin Zou, Lai Wei, Xi Huang, Penghui Zhou

**Affiliations:** 1 State Key Laboratory of Oncology in South China, Collaborative Innovation Center for Cancer Medicine, Sun Yat-sen University Cancer Center, Guangzhou, Guangdong 510060 China; 2 Center for Infection and Immunity, The Fifth Affiliated Hospital, Sun Yat-sen University, Zhuhai, Guangdong 519000 China; 3 Department of Pulmonary and Critical Care Medicine, Fujian Provincial Hospital, Fujian Medical University, Fuzhou, Fujian 350001 China; 4 Infectious Disease Department, The Second Affiliated Hospital of Naval Military Medical University, Shanghai, 200003 China; 5 Huoshenshan Hospital, Wuhan, Hubei 430000 China; 6 The Sixth Affiliated Hospital of Guangzhou Medical University, Qingyuan People’s Hospital, Qingyuan, Guangdong 511500 China; 7 Department of Infectious Diseases, The Third Affiliated Hospital of Sun Yat-sen University, Guangzhou, Guangdong 510630 China; 8 Department of Respiratory and Critical Care Medicine, Fuzhou General Hospital of Fujian Medical University, Fuzhou, Fujian 350025 China; 9 State Key Laboratory of Ophthalmology, Zhongshan Ophthalmic Center, Sun Yat-sen University, Guangzhou, Guangdong 510060 China

**Keywords:** Immunology, Transcription

Dear Editor,

The outbreak of the new coronavirus SARS-CoV-2 has resulted in a global pandemic. Due to the lack of a specific drug against this virus, the current clinical management of this disease mainly depends on supportive care to reduce inflammatory responses and to keep the lung functioning.^1^ Understanding the underlying immunopathology of coronavirus disease 2019 (COVID-19) is therefore of paramount importance for improving the current treatment. In this study, we found a distinct feature of adaptive immunity in severely affected patients, the coincidence of impaired cellular and enhanced humoral immune responses, suggesting that dysregulated adaptive immune responses advanced severe COVID-19. Interestingly, expression of Prothymosin alpha (PTMA), the proprotein of Thymosin alpha-1 (Tα1), was increased in a group of CD8 T memory stem cells accumulated during severe disease. We further showed that Tα1 slightly reduced T cell activation in vitro and promoted proliferation of effector T cells. Moreover, Tα1 treatment relieved the lymphopenia in COVID-19 patients. Our data suggest that early intervention of adaptive immune response might be critical for the prevention of severe COVID-19.

A high rate of severe COVID-19 was reported in immunocompromised patients,^2^ suggesting that an insufficient rather than an overactive antiviral immunity caused this disease. Meanwhile, lymphopenia, a reduction in the number of lymphocytes in the blood, was associated with the severity of COVID-19.^3^ We analyzed the incidence of lymphopenia in 284 patients infected with SARS-CoV-2 (Supplementary information, Table S1), and found that a reduction of lymphocytes was more frequently observed in aged patients except for the group between 0–9 years old who may have an immature immune system (Fig. 1a). These findings denote the pivotal role of the adaptive immunity in the viral clearance and disease control.

**Fig. 1:**
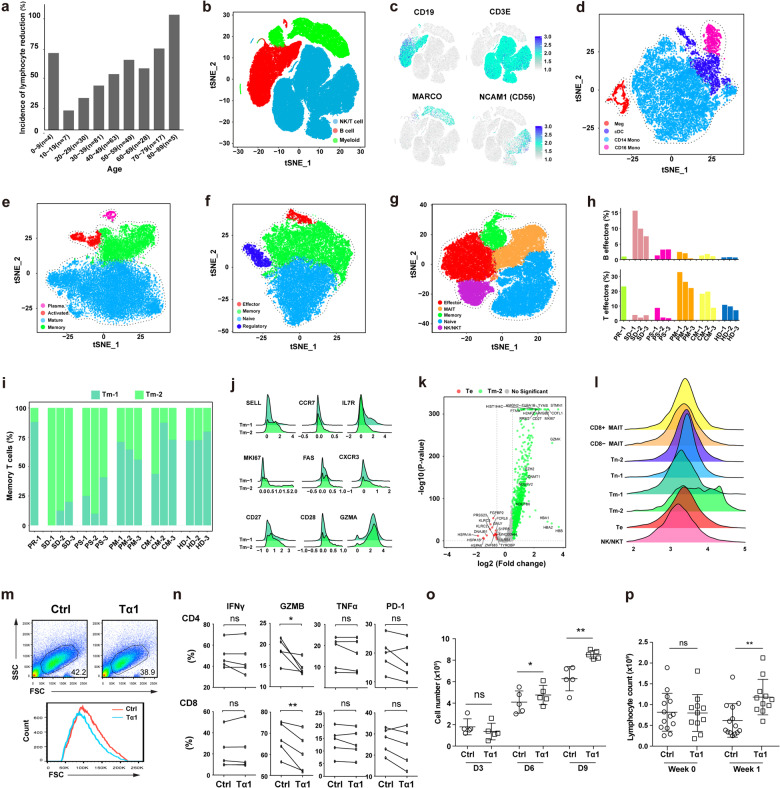
Dysregulated adaptive immune responses in severe COVID-19. **a** Bar plot showing the incidence of lymphocyte reduction in patients of different age groups. **b** The t-SNE plot showing the three main clusters: NK/T cells (blue color), B cells (red color) and myeloid cells (green color). **c** The expression of selected B, T, myeloid and NK cell markers in all cells. The t-SNE plot showing clusters in myeloid cells (**d**), B cells (**e**), CD4 T cells (**f**) and NK/CD8 T cells (**g**). **h** The proportion of B and T effectors in lymphocytes of each patient. **i** The proportion of Tm-1 and Tm-2 in each patient. **j** The ridgeline plot visualizing expression distributions of differentially expressed genes in Tm-1 and Tm-2 cells. **k** Volcano plot showing differentially expressed genes between Te and Tm-2 cells. Red and green dots represent significantly upregulated genes in Te and Tm-2 cells respectively (|log2(FC)| > 0.58, *P* < 0.05). **l** The ridgeline plot visualizing expression distributions of PTMA in NK/CD8 T cell subsets. **m** T cell sizes at day 3 post-activation. **n** Expression of IFNγ, GZMB, TNFα and PD-1 in CD4 and CD8 T cells at day 3 post-activation. **o** T cell numbers on day 3, 6 and 9 post-activation. **p** Lymphocyte counts of SARS-CoV-2 patients treated with or without Tα1. Conventional therapy (Ctrl) *n* = 14, Tα1 treatment *n* = 11, *P < 0.05, **P < 0.01.

In order to understand the immune responses during the disease, we performed single-cell mRNA sequencing (scRNA-seq) of patient peripheral blood mononuclear cells (PBMCs). Thirteen samples were collected from 10 patients at different disease stages, namely pre-severe disease (PR, 1 sample), severe disease (SD, 3 samples), post-severe disease (PS, 3 samples), post-mild disease (PM, 3 samples) and convalescence of mild disease (CM, 3 samples). Four of the enrolled patients experienced severe disease and the other six showed mild symptoms (Supplementary information, Fig. S1a). Three PBMCs from healthy donors (HD) were used as controls.

232 million RNA transcripts in 89,882 cells were obtained after filtering cells with low quality and doublets depletion (Supplementary information, Fig. S1b). A total of 60,406 cells were from COVID-19 patient samples. We then used t-Distributed Stochastic Neighbor Embedding (t-SNE) to visualize the clusters of all the cells identified by a shared nearest neighbor modularity optimization based clustering algorithm implemented in Seurat (Fig. 1b).^4^ Major groups of immune cells in PBMCs, namely myeloid, B and NK/T cells were identified by specific gene expression signatures (Supplementary information, Fig. S1c). NK cells were grouped with T cells. Classic lineage markers further confirmed the identities of these clusters (Fig. 1c). In myeloid cells, four sub-clusters, CD14^+^ monocytes cells (CD14), CD16^+^ monocytes cells (CD16), cDC cells (CD1C), and megakaryocyte cells (CD42a) were identified (Fig. 1d; Supplementary information, Fig. S1e). Increased expression of interferon stimulated genes (IRF7, IFITM1 and IFI6), and HLA genes (HLA-DPB1 and HLA-DRB5) were observed in cDC cells and CD16^+^ monocyte cells. These inflammatory monocytes were observed in all disease stages, indicating their persistent responses to the viral infection (Supplementary information, Fig. S1f). Comparison of samples from the same patient at different disease stages showed a similar trend.

We then focused on adaptive immune cells (CD19^+^, CD79A^+^, CD79B^+^, CD3D^+^, CD3E^+^, and CD3G^+^ cells), and grouped them into B cells, plasma cells, CD4 and NK/CD8 T cells (Supplementary information, Fig. S1d). Given the high levels of NK marker genes (GNLY, NKG7 and FGFBP2) in the CD8 cluster, we named it as NK/CD8 T cells. In order to examine changes of effector cells in different types of adaptive immune cells, we performed separate cluster analysis on the B, CD4 and NK/CD8 T cells. B cells were grouped into 4 major clusters, namely mature, memory, activated B cells and plasma cells (Fig. 1e; Supplementary information, Fig. S1g, h). CD4 T cells consisted of naive, memory, effector and regulatory T cell clusters (Fig. 1f; Supplementary information, Fig. S1j), while clusters of naive, memory, effector (Te), NK/NKT and mucosal-associated invariant T cells (MAIT) were found in CD8 T cells (Fig. 1g; Supplementary information, Fig. S2a). The frequencies of effectors in B cells were significantly increased at the SD stage (Supplementary information, Fig. S1i). Low levels of effectors in CD4 T cells were observed in all of the samples except for one from PR (Supplementary information, Fig. S1k). For CD8 effectors, the frequencies were significant reduced at the SD and PS stages (Supplementary information, Fig. S2b).

Next, we evaluated the humoral and cellular immune responses by measuring the levels of B (plasma cells plus activated B cells) and T effectors (CD4 plus CD8 effectors) in total lymphocytes respectively. Interestingly, a coincidence of high humoral but low cellular immune responses was observed at the SD stage (Fig. 1h). Given the essential role of T cells in viral clearance, a strong antibody response with insufficient T cell support might promote the rapid progression of severe COVID-19 by triggering the antibody-dependent enhancement (ADE) of viral infection.

In order to explore the changes inside the NK/CD8 clusters, we increased the modularity to group more distinct subsets. Two naive, two memory and two MAIT sub-clusters were identified (Supplementary information, Fig. S2c, d). No subsets were detected in the effector and NK/NKT cells. The two sub-clusters of CD8 memory T cells were named as Tm-1 and Tm-2. Significant accumulation of Tm-2 was observed at the SD and PS stages (Fig. 1i). Differential gene expression analysis showed that Tm-2 was a cluster of highly proliferating T memory stem cells (Supplementary information, Fig. S2e). Compared to Tm-1, Tm-2 expressed high levels of T memory stem cell markers (SELL, CXCR3, CCR7, FAS, CD27 and CD28), and the proliferation gene Ki67 (Fig. 1j).^5^ Slightly higher expression of GZMA and lower level of IL7R in the Tm-2 indicated its recent development. The low level of Tm-2 at the PR stage also confirmed its differentiation during severe disease stages (Fig. 1i).

Although both Te and Tm-2 cells were generated during the infection of SARS-COV-2, significant reduction in severe COVID-19 was observed in the Te but not Tm-2 cells. Compared to TM-2, Te cells had higher expression of activation and effector genes (GNLY, NKG7 and GZMH) but almost no expression of Ki67 (Fig. 1k), suggesting that they were terminal-differentiated cells. In contrast, the Tm-2 cells showed high expression of genes involved in epigenetic modification (DNMT1 and EZH2), oxidative phosphorylation (NDUFB6 and NDUFV2), regulation of telomerase, cell cycle and proliferation, etc. These distinct signaling transduction and epigenetic changes might have shaped the stem-like memory phenotype of Tm-2. Interestingly, the expression of PTMA was also significantly increased in Tm-2. Tα1, the first 28 amino acids of PTMA, is involved in T cell development, and has been used for the treatment of certain infection diseases including COVID-19.^6,7^ In addition, Te expressed low level of PTMA, and Tm-2 expressed the highest level of PTMA in all the CD8 T cell sub-clusters (Fig. 1l). We thus suspected that Tα1 might protect T cells during the lymphopenia in COVID-19.

Next, we tested the effect of Tα1 on T cell activation. PBMCs from healthy donors were activated by anti-CD3/CD28 antibodies in vitro with 200 ng/mL Tα1 for 3 days, followed by cultures with IL-2 (200 U/mL) and Tα1 (200 ng/mL) for 6 more days. After 3 days of activation, we found that cell size measured by FSC and SSC was reduced in the group with Tα1 (Fig. 1m), indicating that these T cells were less activated. Tα1 treatment slightly reduced the production of IFNγ and TNFα, although no significant statistical difference was observed (Fig. 1n). Significant reduction of GZMB was observed in both CD4 and CD8 T cells, while the expression of PD-1 was no changed. Compared to the control group, Tα1 had significantly increased T cell numbers at day 6 and 9 although a slight decrease was observed at day 3 (Fig. 1o), indicating that Tα1 promoted the proliferation of effector T cells.

We then analyzed data from 25 severe and critical COVID-19 cases treated at the Huoshenshan Hospital (Wuhan, China) (Supplementary information, Table S1). Of them, 11 patients received daily Tα1 treatment for at least one week, while the other 14 patients were not treated with Tα1 during the hospitalization period. Compared to the non-treated patients, the lymphocyte counts of the treated patients were significantly increased after one week of Tα1 treatment (Fig. 1p; Supplementary information, Fig. S2f). Due to the limited number of patients in this retrospective analysis, we were not able to clearly evaluate the clinical benefits of Tα1 treatment. Nevertheless, our data suggest that the administration of Tα1 could be a potential approach to protect effector T cells during COVID-19.

Consistent with our observation of elevated effector B cells in severely affected patients, high antibody titer was reported to be associated with disease severity and worse outcome both in COVID-19 and SARS,^8–10^ suggesting that ADE accelerated severe disease. The Intravenous Immunoglobulin (IVIg), a polyclonal immunoglobulin G product, can bind the Fc receptors and thus block the ADE of viral infection. Interestingly, a recent report showed that high-dose IVIg reversed the deteriorating course of disease in severe COVID-19 patients.^11^ Taken together, our data suggest that Tα1 and IVIg might be potential approaches for the prevention of severe COVID-19.

## Supplementary information

Supplementary Figures and Methods

Supplementary Table S1
