# Supplementary material for: Dysregulated adaptive immune response contributes to severe COVID-19
Source: Cell Res. 2020 Aug 5;30(9):814–6. doi: 10.1038/s41422-020-0391-9 (PMC7403569; doi:10.1038/s41422-020-0391-9)
Supplement: Supplementary file 1 — Supplementary Figures and Methods [file 41422_2020_391_MOESM1_ESM.pdf]

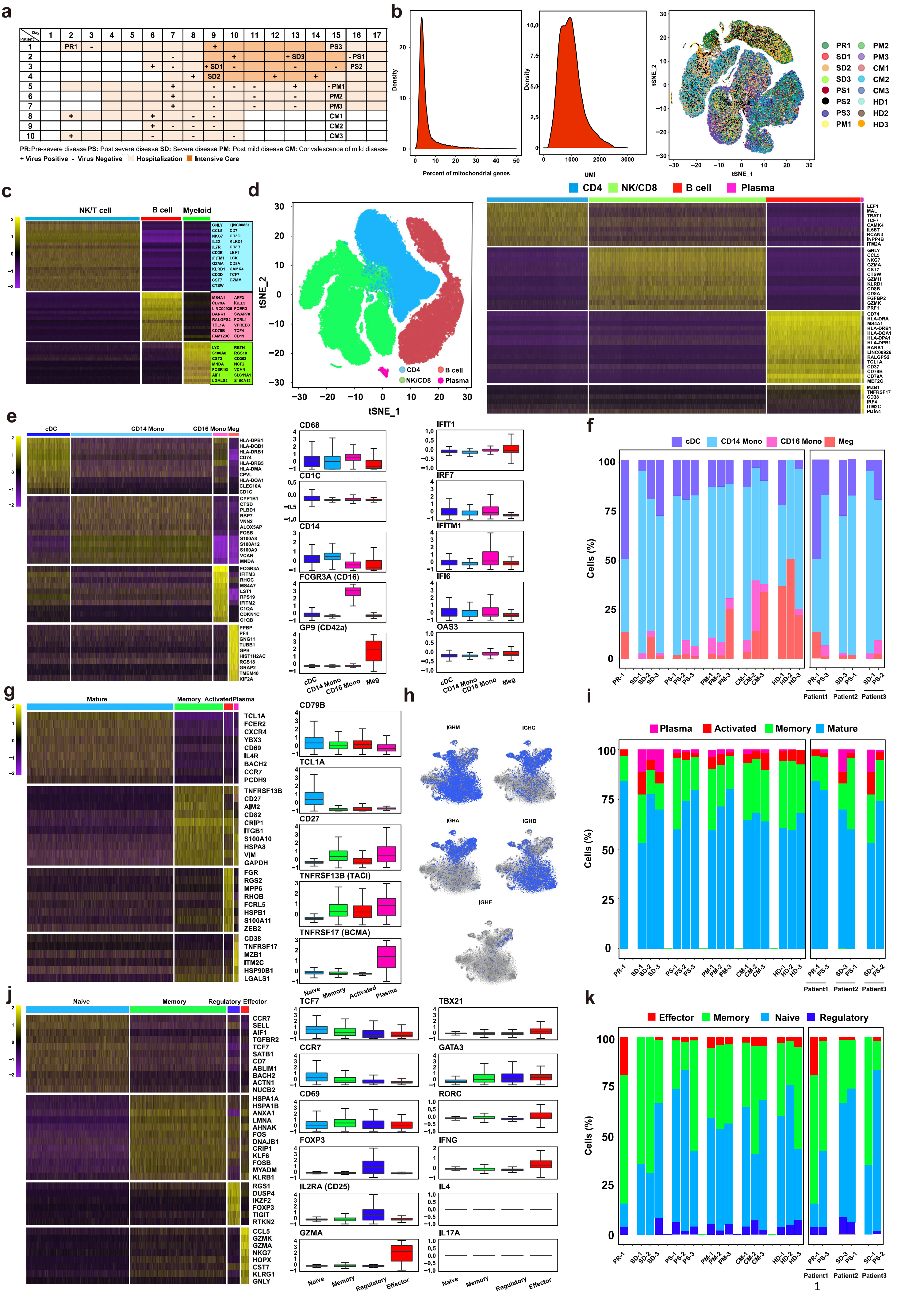
a
Day
Patient
1 2 3 4 5 6 7 8 9 10 11 12 13 14 15 16 17
PR1
PS3
+ SD3
- PS1
+ SD1
PS2
SD2
- PM1
PM2
PM3
CM1
CM2
CM3
PR:Pre-severe disease PS: Post severe disease SD: Severe disease PM: Post mild disease CM: Convalescence of mild disease
+ Virus Positive - Virus Negative Hospitalization Intensive Care
b
Density
Percent of mitochondrial genes
UMI
tSNE_2
tSNE_1
PR1 PM2 SD1 PM3 SD2 CM1 SD3 CM2 PS1 CM3 PS2 HD1 PS3 HD2 PM1 HD3
c
NK/T cell
B cell
Myeloid
d
CD4
NK/CD8
B cell
Plasma
e
cDC
CD14 Mono
CD16 Mono
Meg
CD68
CD1C
CD14
FCGR3A (CD16)
GP9 (CD42a)
IFIT1
IRF7
IFITM1
IFI6
OAS3
f
Cells (%)
Patient1
Patient2
Patient3
g
Mature
Memory
Activated
Plasma
CD79B
TCL1A
CD27
TNFRSF13B (TACI)
TNFRSF17 (BCMA)
Naive
h
IGHM
IGHG
IGHA
IGHD
IGHE
i
Plasma
Activated
Memory
Mature
j
Naive
Memory
Regulatory
Effector
TCF7
CCR7
CD69
FOXP3
IL2RA (CD25)
GZMA
TBX21
GATA3
RORC
IFNG
IL4
IL17A
k
Effector
Memory
Naive
Regulatory

Fig. S1: Sampling time and data quality of scRNA-seq, and features of sub-clusters in myeloid cells, B cells and CD4 T cells.

a. Virus detection result and time of sample collection. **b.** Histograms of percent of mitochondrial genes and UMI distributions (left panel) and the t-SNE plot of cells from all patients after canonical correlation analysis (right panel). **c.** Heatmap showing differentially expressed genes between the three major clusters of PBMCs. The top bar indicates cell types. **d.** The t-SNE projection of the lymphocyte cells, showing 4 sub-clusters including CD4 T cells (blue color), NK/CD8 T cells (green color), B cells (red color) and Plasma cells (purple color) (left panel) and heatmap showing differentially expressed genes between CD4, NK/CD8, B cell and plasma (right panel) **e.** Heatmap showing differentially expressed genes between cDC, CD14 monocytes, CD16 monocytes and megakaryocyte cells (left panel) and box plots showing expression of each gene in these cell clusters (right panel) **f.** The proportion of myeloid subsets (cDC, CD14 monocytes, CD16 monocytes and megakaryocyte cells) in each patient. **g.** Heatmap showing differentially expressed genes between mature, memory, activated B cells and plasma (left panel) and box plots showing expression of each gene in these B cell clusters (right panel). **h.** Expression of signature genes (IGHM, IGHG, IGHA, IGHD and IGHE) in B cells. **i.** The proportion of B cell clusters (Plasma, Activated, Memory, and Mature) in each patient. **j.** Heatmap showing differentially expressed genes between naive, memory, effector and regulatory CD4 T cells (left panel) and box plots showing expression of each gene in these CD4 T cell clusters (right panel). **k.** The proportion of CD4 T cell clusters (Effector, Memory, Naïve and Regulatory) in each patient.

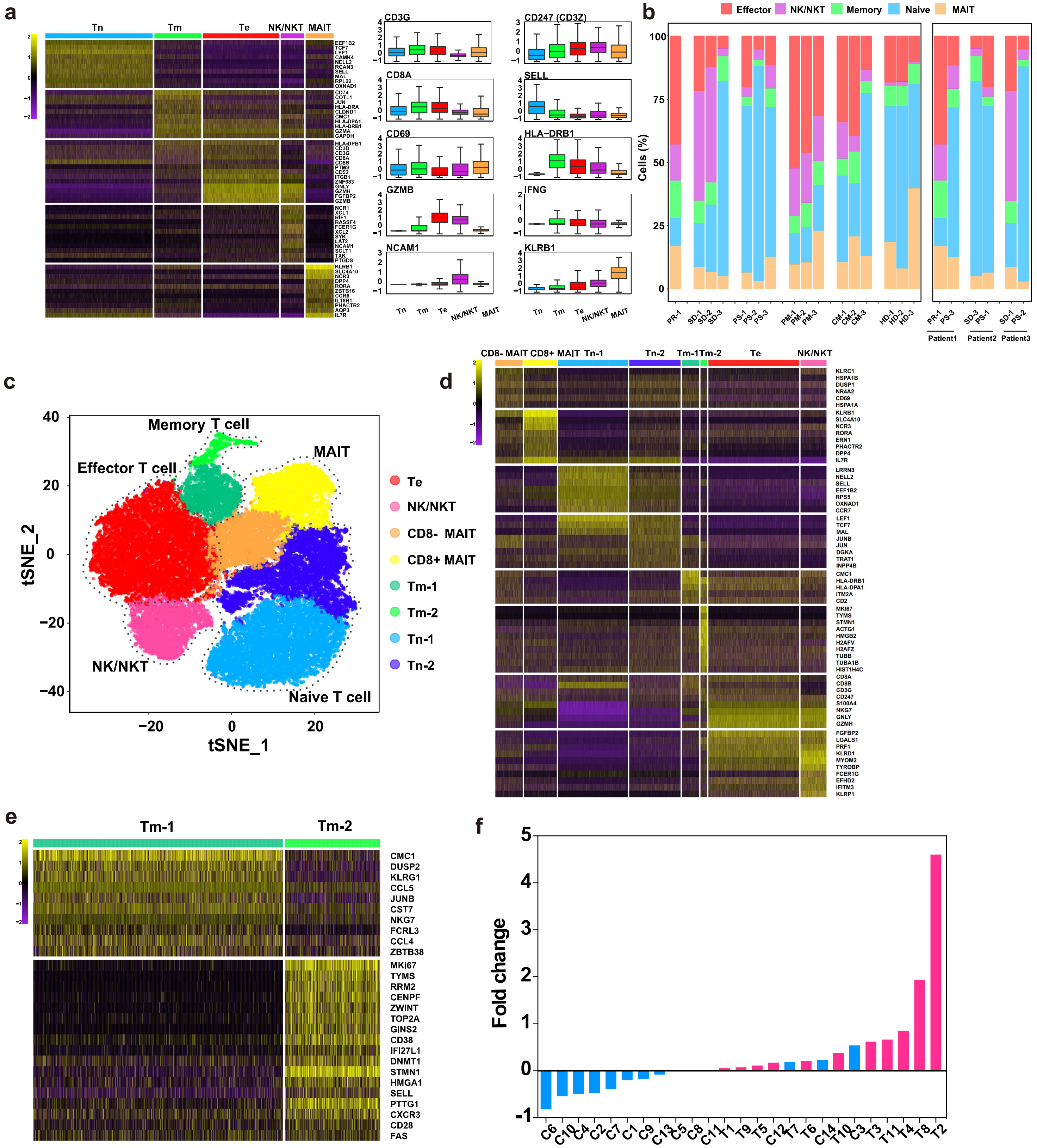
a
Tn
Tm
Te
NK/NKT
MAIT
EEF1B2
TCF7
LEF1
CAMK4
NELL2
RCAN3
SELL
MAL
RPL22
OXNAD1
CD74
COTL1
JUN
HLA-DRA
CLDND1
CMC1
HLA-DPA1
HLA-DRB1
GAPDH
HLA-DPB1
CD3D
CD3G
CD8A
CD8B
PTMS
CD52
ITGB1
ZNF683
GNLY
GZMH
FGFBP2
GZMB
NCR1
XCL1
RASSF4
FCER1G
XCL2
SYK
LAT2
NCAM1
SCLT1
TXK
PTGDS
KLRB1
SLC4A10
NCR3
DPP4
ZBTB16
RORA
CCR6
IL18R1
PHACTR2
AQP3
IL7R
CD3G
CD247 (CD3Z)
CD8A
SELL
CD69
HLA−DRB1
GZMB
IFNG
NCAM1
KLRB1
Tn
Tm
Te
NK/NKT
MAIT
b
Effector
NK/NKT
Memory
Naive
MAIT
Cells (%)
100
75
50
25
0
PR-1
SD-1
SD-2
SD-3
PS-1
PS-2
PS-3
PM-1
PM-2
PM-3
CM-1
CM-2
CM-3
HD-1
HD-2
HD-3
PR-1
PS-3
SD-3
PS-1
SD-1
PS-2
Patient1
Patient2
Patient3
c
Memory T cell
Effector T cell
MAIT
NK/NKT
Naive T cell
tSNE_2
tSNE_1
40
20
0
−20
−40
−20
0
20
Te
NK/NKT
CD8- MAIT
CD8+ MAIT
Tm-1
Tm-2
Tn-1
Tn-2
d
CD8- MAIT
CD8+ MAIT
Tn-1
Tn-2
Tm-1
Tm-2
Te
NK/NKT
KLRC1
HSPA1B
DUSP1
NR4A2
CD69
HSPA1A
KLRB1
SLC4A10
NCR3
RORA
ERN1
PHACTR2
DPP4
IL7R
LRRN3
NELL2
SELL
EEF1B2
RPS5
OXNAD1
CCR7
LEF1
TCF7
MAL
JUNB
JUN
DGKA
TRAT1
INPP4B
CMC1
HLA-DRB1
HLA-DPA1
ITM2A
CD2
MKI67
TYMS
STMN1
ACTG1
HMGB2
H2AFV
H2AFZ
TUBB
TUBA1B
HIST1H4C
CD8A
CD8B
CD3G
CD247
S100A4
NKG7
GNLY
GZMH
FGFBP2
LGALS1
PRF1
KLRD1
MYOM2
TYROBP
FCER1G
EFHD2
IFITM3
KLRP1
e
Tm-1
Tm-2
CMC1
DUSP2
KLRG1
CCL5
JUNB
CST7
NKG7
FCRL3
CCL4
ZBTB38
MKI67
TYMS
RRM2
CENPF
ZWINT
TOP2A
GINS2
CD38
IFI27L1
DNMT1
STMN1
HMGA1
SELL
PTTG1
CXCR3
CD28
FAS
f
Fold change
5
4
3
2
1
0
-1
C6
C10
C4
C2
C7
C1
C9
C13
C5
C8
C11
T1
T9
T5
C12
T7
T6
C14
T10
C3
T3
T11
T4
T8
T2

Fig. S2: Features of sub-clusters in NK/CD8 T cells, and fold change of lymphocyte count in each patient after Tα1 treatment.

a. Heatmap showing differentially expressed genes between naive, memory, effector CD8 T cells, natural killer cells and natural killer T cells (NK/NKT), and mucosal-associated invariant T (MAIT) cells (left panel) and box plots showing expression of each gene in all NK/CD8 T cell clusters (right panel). **b.** The proportion of NK/CD8 T cell clusters (Effector, Memory, Naive, NK/NKT, MAIT) in each patient. **c.** The t-SNE plot of NK/CD8 T cells showing 8 clusters including Te, NK/NKT, CD8+ MAIT, CD8- MAIT, Tm-1, Tm-2, Tn-1 and Tn-2. **d.** Heatmap showing differentially expressed genes between NK/CD8 T cell sub-clusters. **e.** Heatmap showing differentially expressed genes between Tm-1 and Tm-2. **f.** Fold change in lymphocyte count after 1 week of Tα1 treatment in 25 SARS-CoV-2 patients.

Methods

Antibodies and reagents

The antibodies for flow cytometry were purchased from Biolegend or BD. Tα1 (ZADAXIN) was purchased from SciClone Pharmaceuticals. Chromium^{TM} Single cell 5' Library & Gel Bead Kit (1000006), Chromium^{TM} Single cell 3'/5' Library Construction Kit (1000020) was purchased from 10x Genomics. Cytokines were purchased from Peprotech. All cell culture reagents were purchased from Gibco unless otherwise indicated.

Patients and study approval

This study enrolled a total of 10 COVID-19 patients. The 4 patients with severe disease were reported in our previous study[1]. The 6 patients with mild disease (3 at post mild disease stage and 3 at convalescence stage) were recruited from the Fifth Affiliated Hospital (Zhuhai, China) and the Third Affiliated Hospital (Guangzhou, China) of Sun Yat-sen University. Lymphocyte count data from 25 SARS-CoV-2 patients were collected in Huoshenshan hospital (Wuhan, China) by managing doctors. Lymphocyte count data from 256 SARS-CoV-2 patients were collected from designated hospitals in Fujian province during January 3 2020 to march 13 by managing doctors. Informed consent has been obtained from all participated patients. This study received approval from the Research Ethics Committee of the Sun Yat-sen University Cancer Center, the Fifth Affiliated Hospital, the Third Affiliated Hospital of Sun Yat-sen University, the Huoshenshan hospital and the Fujian Provincial Hospital, China (K176-1, HSSLL030, K2020-03-01).

PBMCs Isolation

All procedures were carried out within P2+ laboratory certified for studies of infectious materials. PBMCs were isolated from peripheral venous whole blood samples density gradient cell separation (Ficoll, TBD Science, China), and were preceded for Single cell transcriptome sequencing immediately.

Cell culture

Fresh PBMCs from healthy donors were cultured in IL-2 (200U/ml) containing media in

96-well u-bottomed plates coated with anti-CD3/CD28 (5ug/ml respectively, Peprotech). To test the effect on T cell activation, Tα1 (200ng/ml) was added into T cells culture media while seeding. Cell numbers were recorded at day 3, 6 and 9. Cells harvested after 3 days activation were analyzed by flow cytometry.

Flow cytometry

The antibodies used for cell surface labeling were BUV737 anti-human CD4 (564305, BD), BUV395 anti-human CD8 (563795, BD), FITC anti-human PD-1 (329904, Biolegend). The antibodies used for intracellular staining were PE anti-human IFNG (506507,Biolegend), APC anti-human TNFA (502912, Biolegend), BV421 anti-human Granzyme B (563389, BD). All antibodies for flow cytometry staining were used at 1:200 dilution. Cells were harvested and washed once in 2ml PBS and labeled on ice with indicated antibodies for analysis of surface markers. To determine intracellular cytokine expression, the cells were pre-incubated with Brefeldin A (Biolegend) for 4h before staining. After incubation, cells were stained following surface staining, fixation and permeabilization using the BD Transcription Factor Buffer Set (562574, BD) according to the manufacturer's instructions. All samples were analyzed on a LSRFortessa™ X-20 cell analyzer (BD Biosciences). Analysis of acquired data was performed with the FlowJo software (FlowJo LLC).

Single cell transcriptome sequencing and data preprocessing

The single cell RNA libraries were prepared using the Chromium™ Single cell 3' Reagent Kit of Chromium platform (10x Genomics, USA) following the manufacturer's instruction. Generated single cell RNA libraries were sequenced on the Illumina HiSeq X Ten platform. The CellRanger software (version 3.1.0) was used for preprocessing of the PE150 Illumina sequencing reads. Briefly, raw reads in bcl format were converted to FASTQ format using "cellranger mkfastq", and then the reads in FASTQ format were aligned to human genome reference (hg38, GRCh38) using STAR, and then "cellranger count" was used to derive gene expression matrix for each sample.

Determination of cell types from single cell transcriptome sequencing data

Seurat (v3.1.3) R toolkit was used to analyze the single cell transcriptome sequencing data. Firstly, cells with low quality were filtered out. Briefly, the dead or dying cells with more than 20% mitochondrial RNA content were removed, and the cells with too low number (less than 200) were also removed. Cell doublets were predicted using DoubletFinder. For each patient, a 4% (true) doublet rate was assumed, 5 principal components were used, and the default value of 20% was used for pN (the number of generated artificial doublets expressed as a proportion of the merged real-artificial data). For each library, the PC neighborhood size pK was estimated using as the maxima of the distribution of mean-variance normalized bimodality coefficient scores. Cells expressed more than one marker among the three markers (CD2, CD79A, CD68) were also defined as doublets and removed. Then, the filtered gene expression matrix for each sample was normalized using "NormalizeData" function in Seurat, and only highly variable genes were remained using "FindVariableFeatures" function in Seurat. Next, "FindIntegrationAnchors" and "Integratedata" functions in Seurat were used to integrate the gene expression matrices of all samples, where batch effects between different samples have been adjusted. Next, "RunPCA" function was used to perform the principal component analysis (PCA) and "FindNeighbors" function was used to construct a K-nearest-neighbor graph. Next, the most representative principle components (PCs) selected based on PCA were used for clustering analysis with "FindCluster" function to determine different cell types. Lastly, tSNE was used to visualize the different cell types.

We annotated the cell types using the following rules: Based on the most 10 differentially expressed genes that were derived using "FindAllMarkers" function in Seurat, genes such as CD2, CD3D, CD3E, CD3G and CD247 were used as T cell markers, and genes such as CD19, CD79A, CD79B, BLNK, FCRL5, MS4A1 were used as B cell markers, and genes such as CD14, CD163, CD68, CSF1R, FCGR2A, and CD33 were used as myeloid cell markers. The percentage of CD4 gene expression and CD8A was counted to define CD4+ T cells or CD8+ T cells.

The CD19+, CD79A+, CD79B+, CD3D+, CD3E+, and CD3G+ lymphocytes were further clustered using the single cell analysis pipeline as described above. To get higher resolution clusters, the "resolution" parameter used in FindCluster was set from 0.3 to 0.5.

Differential gene expression analysis between different cell types

Seurat v3 was used to perform differential gene expression analysis between different cell types. For each cell type, DEGs were obtained relative to all of the other cell types using "FindCluster" function in Seurat. DEGs between Tm-2 and Te were obtained using R package edgeR with log2 Fold change > 0.58 and P value < 0.05.

Statistical analysis

All sample sizes were large enough to ensure proper statistical analysis. Statistical analyses were performed using GraphPad Prism (GraphPad Software, Inc.). P values < 0.05 were considered as statistically significant. All t-test analyses were one-tailed t-tests (paired or unpaired depending on the experiments). The number of replicates (n), number of independent experiments performed, and p values for each experiment are reported in the corresponding figure legends.

Data Accession

The raw data files were deposited in the Genome Sequencing Archive of the National Genomics Data Center with the accession number of CRA002572.

Reference

1 Wei L. et al. *SSRN preprint.* (2020).
